# The role of social safety schemas in the persistence of mental health difficulties during adolescence

**DOI:** 10.1111/bjc.12555

**Published:** 2025-06-09

**Authors:** Jenna Alley, Dimitris I. Tsomokos, Summer Mengelkoch, George M. Slavich

**Affiliations:** ^1^ Department of Psychiatry and Biobehavioral Sciences University of California, Los Angeles Los Angeles California USA; ^2^ Department of Psychology and Human Development, UCL Institute of Education University College London London UK

**Keywords:** adolescence, gender differences, longitudinal studies, mental health, social safety

## Abstract

**Background:**

Emotional and behavioural problems (i.e., mental health difficulties and their decomposition into internalizing and externalizing symptoms) often emerge in adolescence and can persist into adulthood if not addressed. Identifying modifiable social‐cognitive processes that influence the persistence of psychopathology across the lifespan is thus essential.

**Method:**

Using data from the Millennium Cohort Study, a nationally representative birth cohort of UK youths born in 2000–2002, we examined whether social safety at age 14 mediated the association between mental health difficulties at age 11 and mental health difficulties at age 17. The sample included 10,782 participants (50% female, 20% non‐White, 21% in poverty).

**Results:**

Mental health difficulties (total symptoms) at age 11 predicted both mental health difficulties at age 17 (*b* = .41, *p* < .001) and negative social safety schemas at age 14 (*b* = .02, *p* < .001). Negative social safety schemas in mid‐adolescence partially mediated the persistence of difficulties from early to late adolescence (*ab* = .01, *p* < .001). In sex‐stratified analyses, we found that negative social safety mediated the persistence of internalizing problems only for females and the persistence of externalizing problems only for males.

**Conclusions and Relevance:**

These findings highlight the important role of social safety schemas in the persistence of adolescent emotional and behavioural problems over time. Based on these results, investments in improving early adolescent mental health by bolstering social safety perceptions may be effective for reducing mental health risks.

**Key points**

- Mental health problems often emerge during adolescence and persist into adulthood if not addressed.
- Identifying modifiable social‐cognitive processes that influence the persistence of psychopathology across life is thus essential.
- We found that negative social safety schemas in middle adolescence mediate the persistence of mental health problems over the course of adolescence.
- We also found that negative social safety schemas mediate the persistence of internalizing problems for females and externalizing problems for males.
- These results highlight the critically important role that social safety schemas play in driving the persistence of adolescent mental health problems over time.

## INTRODUCTION

Adolescence is a sensitive developmental period during which youth face many significant biopsychosocial changes, including a shifting focus from family to friends, increased peer socialization, transitioning to high school, greater risk‐taking, and a wide variety of biological changes due to the pubertal transition (Blakemore & Mills, 2014). Given the substantial social, biological, and personal changes that occur during adolescence, it is not surprising that many mental health difficulties first occur during this time which, if not treated, can persist across the life course (Copeland et al., 2009; Hofstra et al., 2002; Mesman & Koot, 2001; Patalay & Fitzsimons, 2018; Patton et al., 2014; Scardera et al., 2020; Sourander & Helstelä, 2005). This fact becomes particularly important when considered in relation to the current mental health crisis in adolescent populations: According to the Centers for Disease Control and Prevention, four out of 10 high school students in the United States reported feelings of persistent sadness or hopelessness during 2021 and one in six had made a suicide plan (CDC, 2024). Therefore, there is a pressing need to identify modifiable psychosocial factors that may increase risk for mental health difficulties across adolescence.

Past research has shown that the social experiences youth have during adolescence can shape expectations of the social world and future, and can have lasting effects across development, including with respect to mental health (Blakemore & Mills, 2014; Patalay & Fitzsimons, 2018). Specifically, past research has demonstrated that perceived support, or the belief that you can rely on someone, be it friends or family, can positively influence mental health difficulties including depression (Gariépy et al., 2016; Rueger et al., 2016; Santini et al., 2015), as well as internalizing and externalizing symptoms (Mancini et al., 2016; Tandon et al., 2013). Although positive perceptions of social support during adolescence are associated with better mental health outcomes in general, there is evidence that the presence of existing mental health problems can impact how a person perceives and reports on their social support (Gayman et al., 2011; Hafen & Laursen, 2009). Furthermore, this association has been found to differ across the type of mental health difficulty experienced. For example, Hafen and Laursen (2009) found that externalizing symptoms at Grade 6 predicted subsequent degradation in perceived parental support, but internalizing symptoms did not.

Importantly, past studies have found that female adolescents are more likely to experience internalizing symptoms and male adolescents are more likely to experience externalizing symptoms (Leadbeater et al., 1999; Martel, 2013) suggesting that biological sex may be an important consideration in the link between support and mental health, especially across adolescence (Gariépy et al., 2016; Leadbeater et al., 1999; Martel, 2013; Scardera et al., 2020). Further, there also appear to be sex differences in how adolescents perceive and value different types of support as well, with females typically perceiving more social support (Scardera et al., 2020) and valuing family support (Gariépy et al., 2016) more than their male counterparts. In short, although the association between social support and positive health outcomes is relatively consistent, numerous factors impact the strength and even direction of these associations, including existing mental health difficulties and biological sex.

Social Safety Theory (Slavich, 2020) provides one lens through which we can better understand how specific processes related to social support, such as social safety schemas, impact health outcomes in nuanced ways. In brief, Social Safety Theory posits that humans evolved to develop and maintain friendly social bonds that provide critical resources (e.g., food, water, shelter, physical safety) and reduce the risk of potential social conflict and physical injury (Slavich, 2020, 2022; Slavich et al., 2023). Social threats, such as isolation, rejection, and exclusion are, in turn, hypothesized to harm human health insofar as such experiences increase the risk of interpersonal conflict and physical wounding, and can activate components of the immune system involved in inflammation, which promotes a wide variety of somatic and mental health difficulties, including suicidality and depression (Slavich, 2020, 2022; Slavich et al., 2023). Social support is a distinct construct from ones' social safety schemas, but the two are intertwined. Specifically, social experiences across time shape social safety schemas by influencing whether someone generally believes that they live in a socially safe vs. dangerous world.

Although there are numerous factors and experiences that solidify a person's social safety schemas, past experiences with social support are a critical factor (Slavich et al., 2023). From the perspective of Social Safety Theory, those with more positive social safety schemas are expected to experience fewer mental health difficulties across time given that they are equipped with the resources, such as interpersonal support and care, needed to reduce the negative impact of experienced social threat and enhance resilience (Slavich et al., 2023). Ultimately, studying social safety schemas enables us to understand how potentially modifiable beliefs about the availability of safety in the social world impact health, as opposed to focusing on how sources of actual support (e.g., parents, friends) impact health, which are more difficult to modify.

### Present study

Given that cognitive representations of social support can substantially impact mental health, we sought to investigate the potential mediating role of mid‐adolescent *social safety schemas*, as conceptualized by Social Safety Theory (Slavich, 2020), in the association between mental health in early and late adolescence. Given the above‐described research, we reasoned that more positive perceptions of social safety may interrupt the social‐cognitive process through which mental health difficulties occurring in early life persist into adulthood.

To test this possibility, we analysed data from a large, general youth population‐based cohort to longitudinally investigate if social safety schemas at age 14 mediate the association between mental health symptoms at age 11 and 17 in male and female adolescents. Based on the research summarized above, we hypothesized that (a) mental health difficulties, as measured by internalizing and externalizing symptoms, in late childhood to early adolescence (age 11) would persist and predict mental health difficulties in late adolescence (age 17), and that (b) the association between childhood mental health difficulties (age 11) and adolescent mental health (age 17) would be mediated by social safety schemas present in middle adolescence (age 14). If supported, this would highlight the potential utility of positive social safety schemas (e.g., by fostering social warmth, inclusion, and belonging) to reduce the likelihood of significant mental health difficulties persisting across development and impairing psychosocial functioning in late adolescence and into adulthood. Additionally, given prior research documenting substantial sex differences in the presence of internalizing and externalizing symptoms in male vs. female adolescents (Leadbeater et al., 1999; Martel, 2013), we (c) explored sex differences in these associations and hypothesized that female adolescents would exhibit more internalizing problems whereas male adolescents would exhibit more externalizing problems at age 17, although we did not make a priori hypotheses regarding whether the impact of social safety schemas on these outcomes would be sex‐differentiated.

## METHOD

### Participants

Data were derived from the millennium cohort study (MCS), a large, longitudinal birth cohort survey in the United Kingdom tracking ~19,200 children born across the four countries of the UK in 2000–2002 (Joshi & Fitzsimons, 2016), with electoral wards providing the sampling frame (Plewis, 2004). As a nationally representative birth cohort survey, the sampling strategy allowed for representation of families living in neighbourhoods with high levels of child poverty, as well as families in England that lived in areas with a high proportion of ethnic minority populations. Data collection took place through interviews with the mother and using investigator‐based test batteries and additional questionnaires in the child's home. Ethical approvals were obtained in each survey sweep through the National Health Service's Research Ethics Committee system (Shepherd, 2012); consent was given by parents, and youth provided their assent at age 11, and informed consent from 14 years of age onward.

The survey wave at age 14 included 11,717 cohort members who were singletons or first‐born twins or triplets. In this study, we required that cohort members had valid data on both social safety perceptions and internalizing/externalizing problems. Given this condition, 10,782 cohort members (50% female, 20% non‐White, 44% living in disadvantaged areas, 21% in poverty) remained in the analytic sample. Details about the variables used, their frequency plots, and all analyses are in the Supplemental Online Material (SOM, 2024).

### Measures

#### Mental health difficulties (assessed at age 11 and 17)

The primary caregiver completed the Strengths and Difficulties Questionnaire (SDQ) when youth were 11 and 17 years old. The SDQ total difficulties measure (i.e., without the subscale measuring prosociality) has 20 items that factor onto four difficulties subscales: (a) emotion problems, (b) peer problems, (c) conduct problems, and (d) hyperactivity/inattention problems (Goodman, 1997). Youths' mental health difficulties were indicated by the total SDQ score (the sum of these subscales). Each subscale included five items, with responses to each item ranging from 0 (*not true*) to 2 (*certainly true*), with higher values indicating more difficulties (and some items reverse‐scored). The total SDQ difficulties has a maximum score of 40, and a score of ≥17 represents the clinically significant cutoff. In this study, we also scored *internalizing* problems (the sum of emotion and peer difficulties) and *externalizing* problems (the sum of conduct and inattention/hyperactivity). These subscales range from 0 to 20, and have been well‐validated in the literature (A. Goodman et al., 2010). Cronbach's alphas at age 17 were α=.76 for the internalizing and α=.77 for the externalizing scales.

#### Negative social safety (assessed at age 14)

Cohort members responded to three items that assessed their social safety schema: ‘I have family and friends who help me feel safe, secure and happy’; ‘There is someone I trust whom I would turn to for advice if I were having problems’; and, ‘There is no one I feel close to’ (reverse‐scored). The possible responses were: 0 (*agree*), 1 (*neither agree nor disagree*), 2 (and *disagree*). Negative social safety was computed by summing these scores (range: 0–6), with higher values indicating more negative perceptions of social safety (Cronbach's α=.57, a low but acceptable value for a 3‐item scale).

### Covariates (assessed at age 11 or earlier)

#### Neighbourhood disadvantage (survey stratum)

Children's social background was provided by the sampling frame and neighbourhood disadvantage through the Child Poverty Index (CPI). Each UK country has an advantaged and a disadvantaged stratum, where disadvantaged wards were in the upper quartile (poorest 25%) of the CPI. In England, there was a third stratum (ethnic minority) that identified areas with at least 30% ‘Black’ (Black Caribbean, Black African and Black Other) or ‘Asian’ (Indian, Pakistani and Bangladeshi) populations. Area disadvantage and poverty (see below) have both been significantly associated with adolescent mental health and social functioning (Adjei et al., 2024; Hurd et al., 2013; Kim et al., 2022; Wight et al., 2006).

#### Poverty

Poverty is a binary variable corresponding to whether the total household income was below the Organization for Economic Co‐operation and Development (OECD) 60% median indicator. A value of 1 (*yes* indicated that the household's OECD equivalized income was below 60% of the median weekly income as defined by the UK's Department for Work and Pensions national ‘Households Below Average Income’ measure (derived by the MCS).

#### Sex and ethnicity

Information on the youths' biological sex (male or female) was provided by the primary caregiver. Ethnicity was also reported by the primary caregiver, according to the categories of the UK Census at the start of the MCS (i.e., White, Mixed, Indian, Pakistani and Bangladeshi, Black or Black British, Other Ethnic group including Chinese or Other). Race and ethnicity have been associated with both mental health and social support in adolescence (Klineberg et al., 2006; Liang et al., 2016; López et al., 2017).

#### Maternal psychological distress

This assessment was completed by the mother, who was administered the Kessler‐6 (short form) (Mewton et al., 2016) at the age 11 wave, with a total score from 0 to 24 (higher scores indicate greater level of psychological distress). Maternal psychological distress at baseline was used instead of paternal distress because the primary adult respondent in MCS was the mother, and as a result, there were more data for mothers than fathers. In addition, the risk of youth psychopathology is higher for those with maternal than paternal mental health difficulties (Ayano et al., 2021; Rajyaguru et al., 2021).

#### Verbal ability

Cognitive ability in MCS was assessed with measures from the British Ability Scales (BAS) II (Hill, 2005). At 11 years old, the BAS Verbal Similarities test was administered by a trained assessor, measuring verbal reasoning and linguistic knowledge. The age standardized T‐score (using exact age) for this assessment was a numerical variable ranging from 20 to 80. Cognitive ability (and verbal ability, in particular) has been associated with both adolescent mental health and social competence (Botting & Conti‐Ramsden, 2008; Cohen et al., 2013; Dall et al., 2022; von Stumm et al., 2023).

### Data analysis

#### Preliminary analysis: Missingness, sample bias, and correlations

Descriptive analyses were performed to identify any differences between participants in the analytic sample and those excluded from it, and to ensure that missingness was both low and data was not Missing Completely at Random, an analysis which also informed the imputation process. Pairwise correlations were calculated between the numerical variables.

#### Mediation model

**FIGURE 1:**
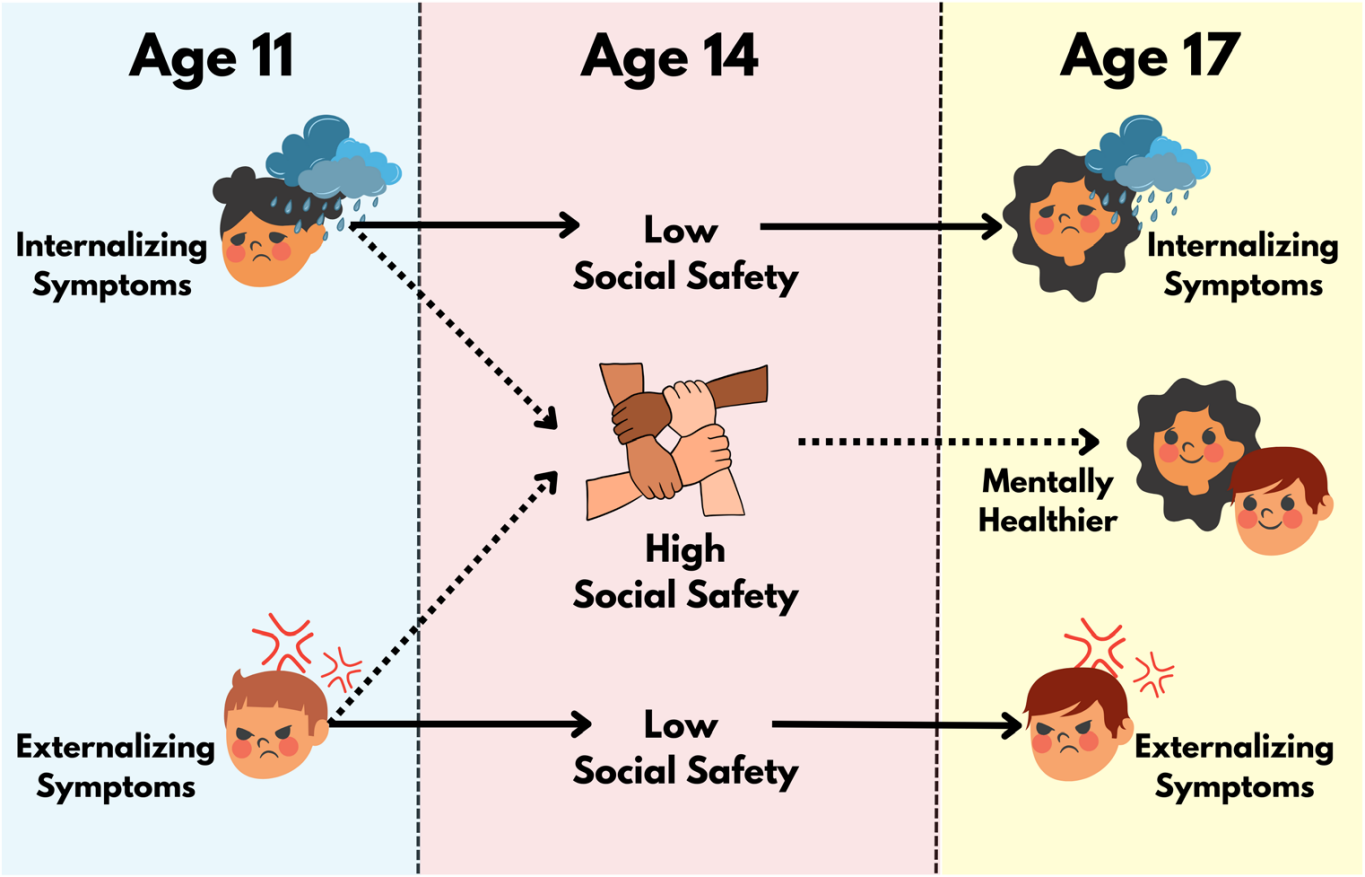
Graphical depiction of sex‐stratified analysis results. For females, internalizing symptoms at age 11 persisted more strongly to age 17 when perceptions of social safety at age 14 were low. In contrast, for males, externalizing symptoms at age 11 persisted more strongly to age 17 when perceptions of social safety at age 14 were low. For both sexes, high levels of social safety at age 14 predicted better mental health at age 17.

We used a structural equation model to test the hypothesis that social safety schemas in middle adolescence (age 14) mediated the association between mental health difficulties in early adolescence (age 11) and late adolescence (age 17). First, this was done in a minimally adjusted model, controlling for biological sex, poverty, and neighbourhood disadvantage. The data were then refitted in a fully adjusted model, which additionally controlled for the youth's ethnicity and verbal ability, as well as the mother's psychological distress. The models were fitted both before and after data imputation. In a secondary analysis, the specificity of the mental health difficulties was tested by delineating total mental health difficulties into the internalizing and externalizing problem subscales. Given that there were now two outcomes, we applied a Bonferroni correction and considered findings to be significant only when the *p*‐value was conservatively set to *p* < .025. Finally, in a sex‐stratified analysis, graphically represented in Figure 1, these models were refitted separately for male and female cohort members. All models were weighted based on the MCS survey UK‐wide design characteristics. Complete details of this analysis with output for all models (unstandardized and standardized coefficients, confidence intervals, etc.) are presented in the Supplemental Online Material (SOM, 2024).

#### Imputation method

Missing data were imputed using multiple imputation by chained equations (Raghunathan et al., 2000), and the imputed datasets were combined following Rubin's rules (Rubin, 1987). All calculations were performed using R (R Core Team, 2021) with the ‘mice’ (van Buuren & Groothuis‐Oudshoorn, 2011) and ‘lavaan’ (Rosseel, 2012) packages.

## RESULTS

Characteristics of the analytic sample (*N* = 10,782) are presented in Table 1, and the main results of the mediation analysis are in Table 2. As hypothesized, mental health difficulties at age 11 significantly predicted both negative social safety schemas at age 14 years (*b* = .02, *p* < .001) and mental health difficulties at age 17 (*b* = .41, *p* < .001). Moreover, negative social safety schemas at age 14 years partially mediated the association between mental health difficulties from early (11 years) to late (17 years) adolescence (*ab* = .01, *p* < .001), even after full adjustments with confounders in a survey‐weighted, imputed, saturated mediation model. Notably, the prospective association between negative social safety schemas at age 14 and subsequent mental health difficulties at age 17 (standardized *β* = .07, *p* < .001) was comparable in effect size to that of family poverty (*β* = .05, *p* < .001), with these being the two strongest predictors of mental health difficulties in late adolescence. In a secondary analysis, we found the same effect for internalizing and externalizing problem scores; in each case, prior internalizing and externalizing problems predicted later internalizing and externalizing problems, respectively, and these effects were partially mediated through negative social safety schemas in middle adolescence (see Table 2).

**TABLE 1:** Participant demographics. Characteristics of the analytic sample and sample bias analysis against the rest of the sample (i.e., excluded participants).

Characteristic	Analytic sample (*N* = 10,782)	Rest of sample (*N* = 935)	*p*‐value^a^
**Sex, *n* (%)**			**<.001**
Male	5350 (50)	529 (57)	
Female	5432 (50)	406 (43)	
**Neighbourhood disadvantage, *n* (%)**			**<.001**
England – advantaged	2995 (28)	242 (26)	
England – disadvantaged	2641 (24)	233 (25)	
England – ethnic	1411 (13)	150 (16)	
Wales – advantaged	519 (4.8)	23 (2.5)	
Wales – disadvantaged	1016 (9.4)	110 (12)	
Scotland – advantaged	644 (6.0)	44 (4.7)	
Scotland – disadvantaged	534 (5.0)	40 (4.3)	
Northern Ireland – advantaged	431 (4.0)	26 (2.8)	
Northern Ireland – disadvantaged	591 (5.5)	67 (7.2)	
**Ethnicity, *n* (%)**			**<.001**
White	8546 (80)	418 (74)	
Mixed	511 (4.8)	23 (4.0)	
Indian	288 (2.7)	15 (2.6)	
Pakistani and Bangladeshi	762 (7.1)	64 (11)	
Black or Black British	329 (3.1)	34 (6.0)	
Other Ethnic group (INC Chinese, Other)	263 (2.5)	14 (2.5)	
(Missing)	83	367	
**Poverty, *n* (%)**			**<.001**
0 (Not in poverty)	7793 (79)	491 (63)	
1 (In poverty)	2108 (21)	289 (37)	
(Missing)	155	881	
**Maternal education, *n* (%)**			.**025**
0 (No university degree)	8768 (92)	710 (94)	
1 (University degree)	799 (8.4)	45 (6.0)	
(Missing)	1215	180	
**Maternal distress, mean (*SD*)**	3.9 (4.3)	4.9 (4.8)	**<.001**
(Missing)	1394	243	
**Verbal ability, mean (*SD*)**	59 (10)	55 (12)	**<.001**
(Missing)	1006	214	
**Negative social safety schemas, age 14, mean (*SD*)**	.52 (.92)	.47 (.90)	.16
(Missing)	0	598	
**Internalizing problems, age 11, mean (*SD*)**	3.1 (3.1)	4.3 (3.9)	**<.001**
(Missing)	824	163	
**Externalizing problems, age 11, mean (*SD*)**	4.3 (3.5)	5.9 (4.2)	**<.001**
(Missing)	846	163	
**Internalizing problems, age 17, mean (*SD*)**	3.7 (3.4)	5.2 (4.2)	**<.001**
(Missing)	2666	410	
**Externalizing problems, age 17, mean (*SD*)**	3.5 (3.2)	5.2 (4.0)	**<.001**
(Missing)	2667	411	

^a^ Pearson's Chi‐squared test; Welch Two Sample *t*‐test.

**TABLE 2:** Coefficients for direct and indirect effects in the saturated, fully adjusted mediation model for all participants (*N* = 10,782, imputed, survey‐weighted).

	SDQ (age 17)	Internalizing (age 17)	Externalizing (age 17)	Negative social safety (age 14)
	Unstandardized *b* (std. err.)			
SDQ (age 11)	.41 (.02)***	–	–	.02 (.00)***
Internalizing (age 11)	–	.35 (.02)***	–	.02 (.00)***
Externalizing (age 11)	–	–	.37 (.02)***	.02 (.00)***
Negative social safety (age 14)	.40 (.08)**	.25 (.06)***	.17 (.05)***	
Sex (ref male)	−.42 (.14)**	−.60 (.09)***	.23 (.09)**	
Poverty (age 11)	.65 (.22)**	.34 (.14)*	.37 (.13)**	
Maternal distress (age 11)	.03 (.02)	.04 (.01)***	.01 (.01)	
Verbal ability (age 11)	−.02 (.01)*	−.01 (.01)*	−.01 (.01)*	
	Indirect effects (*ab*)			
SDQ → NSS → SDQ	.01 (.00)***			
INT → NSS → INT		.01 (.00)***		
EXT → NSS → EXT			.00 (.00)**	

*Note:* Coefficients for neighbourhood disadvantage and ethnicity are not included here for brevity, but can be found in the Supplemental Online Material, along with the exact *p*‐values and 95% confidence intervals for all results.

Abbreviations: EXT, externalizing problems; INT, internalizing problems; SDQ, Strengths and Difficulties Questionnaire.

* *p* < .05.

** *p* < .01.

*** *p* < .001 for two‐sided Wald z‐test.

### Sex‐stratified analyses

In sex‐stratified analyses, we confirmed the main finding for both males and females—namely, early‐adolescence mental health difficulties (age 11) significantly predicted late‐adolescence mental health difficulties (age 17) for each sex separately, and these associations were mediated by the development of negative social safety schemas (at age 14) (see Tables 3 and 4). When considering internalizing and externalizing problems separately—and while correcting for multiple comparisons – we found that negative social safety at age 14 partially mediated the association between early (age 11) and late (age 17) internalizing problems for female (*ab* = .005, *p* = .022) but not male (*ab* = .004, *p* = .052) adolescents. Conversely, negative social safety schemas (age 14) partially mediated the association between early (age 11) and late (age 14) externalizing problems only for male (*ab* = .005, *p* = .022) but not female (*ab* = .004, *p* = .045) adolescents.

**TABLE 3:** Coefficients for direct and indirect effects in the saturated, fully adjusted mediation model for female participants only (*N* = 5432, imputed, survey‐weighted).

	SDQ (age 17)	Internalizing (age 17)	Externalizing (age 17)	Negative social safety (age 14)
	Unstandardized *b* (std. err.)			
SDQ (age 11)	.43 (.03)***	–	–	.01 (.00)***
Internalizing (age 11)	–	.37 (.02)***	–	.02 (.01)*
Externalizing (age 11)	–	–	.37 (.02)***	.02 (.01)**
Negative social safety (age 14)	.48 (.12)***	.33 (.07)***	.17 (.06)**	
Poverty (age 11)	.98 (.29)***	.53 (.17)**	.52 (.17)**	
Maternal distress (age 11)	.05 (.03)	.06 (.01)***	.01 (.01)	
Verbal ability (age 11)	−.02 (.01)	−.01 (.01)	−.01 (.01)	
	Indirect effects			
SDQ → NSS → SDQ	.**01 (.00)** **			
INT → NSS → INT		.**01 (.00)** *		
EXT → NSS → EXT			.00 (.00)*	

*Note*: Coefficients for neighbourhood disadvantage and ethnicity are not included here for brevity, but can be found in the Supplemental Online Material, along with the exact *p*‐values and 95% confidence intervals for all results. A Bonferroni correction has been applied to the focal coefficients of the two models in this table and the bold values for the indirect effects indicate that they retained significance after this correction (*p* < .025).

Abbreviations: EXT, externalizing problems; INT, internalizing problems; SDQ, Strengths and Difficulties Questionnaire.

* *p* < .05.

** *p* < .01.

*** *p* < .001 for two‐sided Wald z‐test.

**TABLE 4:** Coefficients for direct and indirect effects in the saturated, fully adjusted mediation model for male participants only (*N* = 5350, imputed, survey‐weighted).

	SDQ (age 17)	Internalizing (age 17)	Externalizing (age 17)	Negative social safety (age 14)
	Unstandardized *b* (std. err.)			
SDQ (age 11)	.41 (.03)***	–	–	.02 (.00)***
Internalizing (age 11)	–	.34 (.03)***	–	−.01 (.00)***
Externalizing (age 11)	–	–	.39 (.03)***	.03 (.01)***
Negative social safety (age 14)	.32 (.12)**	.16 (.07)*	.19 (.07)**	
Poverty (age 11)	.41 (.34)	.22 (.20)	.24 (.21)	
Maternal distress (age 11)	.00 (.03)	.03 (.02)	.00 (.02)	
Verbal ability (age 11)	−.03 (.01)*	−.02 (.01)**	−.01 (.01)	
	Indirect effects			
SDQ → NSS → SDQ	.**01 (.00)** *			
INT → NSS → INT		.00 (.00)		
EXT → NSS → EXT			.**01 (.0)** *	

*Note*: Coefficients for neighbourhood disadvantage and ethnicity are not included here, but can be found in the Supplemental Online Material, along with the exact *p*‐values and 95% confidence intervals for all results presented. A Bonferroni correction has been applied to the focal coefficients of the two models in this table and bold values for indirect effects indicate that they remained significant after this correction (*p* < .025).

Abbreviations: EXT, externalizing problems; INT, internalizing problems; SDQ = Strengths and Difficulties Questionnaire.

* *p* < .05.

** *p* < .01.

*** *p* < .001 for two‐sided Wald z‐test.

## DISCUSSION

The present study investigated if perceptions of social safety influence the persistence of mental health problems across adolescence. Consistent with prior research (Blakemore & Mills, 2014; Copeland et al., 2009; Hofstra et al., 2002; Mesman & Koot, 2001; Patalay & Fitzsimons, 2018; Patton et al., 2014; Scardera et al., 2020; Sourander & Helstelä, 2005), we found that mental health difficulties in early adolescence significantly predicted mental health difficulties in late adolescence in a large, nationally representative, population‐based cohort study. Beyond replicating prior studies, these findings extend past research by showing that negative social safety schemas mediate the association between earlier and later mental health difficulties – for both internalizing and externalizing problems – even after adjusting for a wide variety of potential confounds. Therefore, improving social safety schemas in middle adolescence may be one way to help prevent mental health difficulties from persisting across adolescence and into young adulthood.

In sex‐stratified analyses, we found that the associations observed differed across the biological sexes. Consistent with prior research (Leadbeater et al., 1999; Martel, 2013), females and males differed in how social safety schemas impacted the presence of internalizing vs. externalizing symptoms at age 17. More specifically, for female adolescents, while the direct paths from mental health difficulties at 11 years to mental health difficulties at 17 years were significant in all cases (i.e., total problems, internalizing problems, and externalizing problems), this was not the case for the indirect paths. After applying a Bonferroni correction, the indirect paths from mental health difficulties at age 11 to mental health difficulties at age 17 through social safety schemas at age 14 were only significant for internalizing and total problems, but not for externalizing problems. Again, these results point to the differences in the prevalence of certain types of mental health difficulties across the biological sexes and provide evidence for the unique role that social safety may play in this context.

By contrast, for males, we found that – after a Bonferroni correction – the indirect paths from mental health difficulties at age 11 to mental health difficulties at age 17 through social safety schemas at age 14 were only significant for externalizing and total problems, but not for internalizing problems. We also note that, for female (but not male) adolescents, poverty significantly predicted all three outcomes indexing mental health at age 17, with those experiencing poverty faring worse than those not experiencing poverty. This finding suggests that, although it may be health promoting to increase female adolescents' positive perceptions of social safety, other factors such as poverty must also be considered. Ultimately, the specificity provided by our sex‐stratified analyses may help clinicians determine if an intervention targeting social safety schemas is likely to be impactful based on a client's biological sex, socio‐economic status, and specific symptom profile.

Broadly, these effects have important implications for clinical practice and public health interventions aiming to combat anxiety, depression, inattention, and peer problems in adolescents. First, the results suggest that bolstering youths' perception of social safety as they transition into late adolescence may be one way to reduce persistent mental health difficulties, which can be targeted by clinicians today. Based on the fully adjusted model controlling for a range of socio‐economic, family, and individual confounders, a one‐unit improvement in social safety for females at age 14 was associated with a .5‐unit improvement in the total SDQ score, whereas for males, it was associated with a .3‐unit improvement in the total SDQ score. Therefore, maximally improving social safety schemas could lead to a 2‐point reduction in the total SDQ score for females and a 1.2‐point reduction for males. Given that the well‐established SDQ clinical cutoff score (indicating the likely presence of psychopathology) is 17 (Bryant et al., 2020), such reductions, although they may seem small at face value, have the potential to be clinically significant when scaled up to population levels (Carey et al., 2023) considering that the median SDQ score in our sample for those in the clinically relevant range was 19. In practice, this implies that the small‐but‐significant effects reported here could be clinically relevant, especially for female adolescents.

### Strengths and limitations

The study has several strengths. First, we used a large, longitudinal dataset with a diverse population, which enabled us to characterize temporal associations between mental health in early adolescence, social safety schemas, and mental health in late adolescence. Second, we specified the unique influence that social safety has on different dimensions of mental health. Finally, we estimated these effects separately for males and females.

Several limitations should also be noted. First, our measure of social safety schemas included only three items and may not be as comprehensive as theoretical work suggests (Slavich et al., 2023). Second, we could not account for other factors that may impact social safety, such as early adversity, personality, or genetics (Slavich et al., 2023). Third, additional cross‐cultural research is needed to examine the generalizability of these findings to other nations, communities, and contexts. Fourth, causality cannot be assumed due to potentially unmeasured confounders. Finally, although significant, the effect sizes reported here are relatively small and future research should examine their clinical relevance in greater detail.

## CONCLUSION

In conclusion, the present results highlight the important role that social safety schemas play in driving the persistence of adolescent mental health difficulties over time. In addition, the data extend existing research by showing that prior mental health difficulties predict subsequent difficulties both directly and indirectly through adolescents' perceptions of social safety. Based on these results, public health efforts aimed at reducing adolescent psychopathology are likely to benefit from fostering social connection, inclusion, and belonging (Slavich et al., 2022). The data also provide practitioners with an avenue for fostering psychosocial resilience. Indeed, investments in improving early adolescent mental health by bolstering social safety may be useful for helping improve the health and well‐being of our youth, who are presently experiencing a mental health crisis (CDC, 2024).

## AUTHOR CONTRIBUTIONS

**Jenna Alley:** Conceptualization; writing – original draft; writing – review and editing; project administration; methodology. **Dimitris I. Tsomokos:** Conceptualization; writing – original draft; writing – review and editing; formal analysis; methodology. **Summer Mengelkoch:** Writing – review and editing; conceptualization. **George M. Slavich:** Writing – review and editing; supervision; conceptualization; funding.

## FUNDING INFORMATION

J.A., S.M., and G.M.S. were supported by grant #OPR21101 from the California Governor's Office of Planning and Research/California Initiative to Advance Precision Medicine. D.I.T. was supported by Alphablocks Nursery School. The findings and conclusions in this article are those of the authors and do not necessarily represent the views or opinions of these organizations, which had no role in designing or planning this study; in collecting, analysing, or interpreting the data; in writing the article; or in deciding to submit this article for publication.

## CONFLICT OF INTEREST STATEMENT

The authors declare no conflicts of interest with respect to this work.

## Data Availability

The data that support the findings of this study are publicly available from the UK Data Service: http://doi.org/10.5255/UKDA‐Series‐2000031.
